# Developing Crash Severity Model Handling Class Imbalance and Implementing Ordered Nature: Focusing on Elderly Drivers

**DOI:** 10.3390/ijerph18041966

**Published:** 2021-02-18

**Authors:** Seunghoon Kim, Youngbin Lym, Ki-Jung Kim

**Affiliations:** 1 City and Regional Planning, Knowlton School, The Ohio State University, Columbus, OH 43210, USA; gaeguri10@gmail.com; 2 Department of Smart Car Engineering, Doowon Technical University, Paju 10838, Korea

**Keywords:** older drivers, machine learning, cost-sensitive learning, ordered nature, crash severity

## Abstract

Along with the rapid demographic change, there has been increased attention to the risk of vehicle crashes relative to older drivers. Due to senior involvement and their physical vulnerability, it is crucial to develop models that accurately predict the severity of senior-involved crashes. However, the challenge is how to cope with an imbalanced severity class distribution and the ordered nature of crash severities, as these can complicate the classification of the severity of crashes. In that regard, this study investigates the influence of implementing ordinal nature and handling imbalanced class distribution on the prediction performance. Using vehicle crash data in Ohio, U.S., as an example, the eight machine learning classifiers (logistic and ordered logistic regressions and random forest and ordered random forest with or without handling imbalanced classes) are suggested and then compared with their respective performances. The analysis outcomes show that balancing strategy enhances performance in predicting severe crashes. In contrast, the effects of implementing ordinal nature vary across models. Specifically, the ordered random forest classifier without balancing appears to be superior in terms of overall prediction accuracy, and the ordered random forest with balancing outperforms others in predicting severer crashes.

## 1. Introduction

As to demographic shifts characterized by aging phenomena [1,2], the risks of vehicle accidents involving older drivers have been increasing. They have also been gaining broader attention along with an increase in life expectancy due to the development of technologies as well as the pursuit of quality of life [2,3,4]. For example, according to the 2017 National Population Projection [5], the proportion of 70 years and over age cohort grows from 11.43% in 2020 to 16.74% in 2040 in the United States. The number of licensed older drivers (70 and older) has increased by 65 percent from 1997 to 2018. Moreover, the proportion of licensed drivers aged 70 years and over has also increased by 10% between 1997 (73%) and 2018 (83%).

Regardless of the increase in the number of older drivers along with the aged population, the vehicle crash involvement of older drivers has decreased. However, the injury risks of the older driver-involved accidents are higher than that of other accidents [6]. For instance, fatalities of older drivers and their passengers are higher than other types of crash fatalities [7,8,9,10]. According to the Insurance Institute for Highway Safety (2020) [4], drivers 80 years and older are likely to be killed by 0.658% in vehicle accidents, which is approximately 4.8 times higher than those of younger cohorts (0.137% for aged 30–39). Therefore, we can expect that both policymakers and transportation safety planners will soon face the problem of senior-related traffic accidents (e.g., a surge of senior driver crashes or an increased risk of senior driver-related crash severities). 

This study aims to predict the vehicle crash severity (i.e., injury level) caused by senior drivers. Previously, a few scholars have investigated the senior involved traffic accidents [3,6,8,11,12,13]. Most studies addressed that older drivers are more susceptible to being injured and killed by accidents. For example, Hanrahan et al. (2009) [3] investigated the association between driver age and crash severity in Wisconsin using logistic regression. They found a strong positive association between the age of drivers and crash severity so that an accident induced by older drivers has higher risks of severe injury and fatality. 

We argue that there are two major issues in crash severity analysis. Firstly, most classification problems of crash severity are subject to imbalanced datasets for their analytic investigation. That is, minority classes (severe and fatal crashes) are likely to be overwhelmed by majority classes (i.e., possible injuries or property damage only crashes). Thus, the classifiers tend to predict majority classes more accurately than minority counterparts [14,15]. As Fiorentini and Losa (2020) [16] pointed out, most research works predicting crash severity overlooked the imbalanced class problem, leading them to develop and compare crash severity prediction models with or without handling the imbalanced problem. The authors recommended addressing the imbalanced issue when predicting crash severity. 

Moreover, Mafi et al. (2018) and Al Mamlook et al. (2020) [12,13] applied multiple machine learning algorithms to analyze the senior driver-related accidents utilizing the cost-sensitive classifiers to solve the class imbalance problem. Mafi et al. (2018) [12] produced the models that predict severer injuries better using cost-sensitive learning. They concluded that the random forest cost-sensitive classifier is the best model in predicting injury severity compared to instance-based and C4.5 models. In the same vein, Al Mamlook et al. (2018) [13] developed several machine learning algorithms with cost-sensitive learning schemes (e.g., Synthetic Minority Oversampling Technique, SMOTE). The authors concluded that random forest and the light gradient boosting algorithm are the best classifiers. They also clarified that the most influential risk factors are age, car age, and traffic volume. 

Secondly, since the response variable of crash severity analysis has an ordered nature [17], it is rational to account for the ordinal structure of the data in crash severity modeling, which can also improve the efficacy of the models [18]. Thus, conventional ordered probability models have been widely adopted within the literature. However, due to the way that predictors affect outcome probabilities, the ordered models are not always superior to the unordered counterparts [19]. In other words, a tradeoff has inherently to be made between recognizing the ordering of the responses and losing the flexibility in the model specification [19]. Therefore, to find the best model in predicting crash severity outcomes, it is reasonable to develop and compare ordered and unordered models. For instance, Zhang et al. (2018) [20] compare the performance in predicting crash severity among ordered probit and multinomial logistic regression models against machine learning algorithms without considering an ordinal structure. The result shows that machine learning approaches outperform the conventional modeling frameworks. 

As previously discussed, older drivers are more vulnerable than their younger counterparts, so that it is more crucial to appropriately predict the severe outcomes of older driver-related vehicle crashes. Although previous literature emphasizes the importance of handling imbalanced class distribution as well as accounting for ordered nature of crash severity, there is a lack of research works to take both features into consideration to the crash modeling procedure. Hence, this study attempts to fill these knowledge gaps in the existing literature. Specifically, it contributes to developing and comparing respective performances of multiple predictive models that evaluate the effects of adequately handling imbalanced classes in the response and/or addressing ordered nature in the severity of crashes pertinent to older drivers. In that regard, we ask the following research questions: (1) will ordered machine learning (ML) algorithms outperform unordered ML counterparts in terms of predictive performance? (2) What is the contribution of handling imbalanced class to the predictive performances among ML models? and (3) what are the influential (contributing) factors in classifying severity outcomes of vehicle crashes by senior drivers?

To answer these questions, we have selected various learning algorithms: the multinomial and ordered logistic regression algorithms are based on the log-linear relationship between predictors and outcome variables (i.e., parametric), while multinomial and ordered random forest do not assume any linear relationship between them (i.e., non-parametric). We attempt to explore the heterogeneous influences of addressing ordered nature of crash severity and balancing strategy on the prediction performances across models. For example, implementing a balancing strategy can raise the prediction performance of parametric models while this may not be so effective as non-parametric counterparts, or it can make no difference between ordered models (e.g., ordered logistic model and ordered random forest). 

Hence, based on the comparison of the predictive performances of eight classifiers, we can evaluate the influences of addressing an ordinal nature and balancing strategy into various models on the prediction capability of crash severity outcomes by older drivers. We also compare the risk factors of eight classifiers and discuss how both features affect those classifiers to identify influential risk factors. 

The rest of this paper is structured as follows. Section 2 explains the analytic operational frameworks and data adopted for this study. Section 3 illustrates the outcomes of our selected models and discusses them in detail, followed by Section 4 that suggests relevant policy considerations and summarizes this research. 

## 2. Methodology

### 2.1. Research Framework

**Figure 1:**
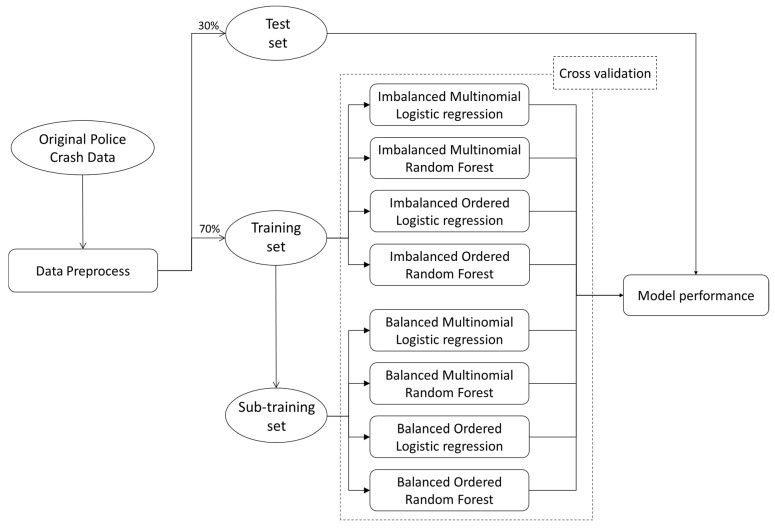
Research framework.

Figure 1 shows the overall analytic operational process adopted in this study. We preprocess the original datasets by eliminating outliers and unnecessary cases. The dataset is randomly partitioned into training (70%) and test sets (30%) thereafter. As a further step (for learning algorithms by means of balanced datasets), the training set is split into the sub-training set (the balanced sub-training set). Different statistical and machine learning models were trained under original training (i.e., four models using imbalanced class) and balanced sub-training sets, respectively. Then, those eight classifiers learned from both training sets are employed to predict the crash severity for the test set. The performance of eight classifiers is estimated and compared, followed by the identification and comparison of the ten most influential factors of each model. 

### 2.2. Data Description

This research is based on the actual vehicle crash data obtained from the Ohio Department of Public Safety (ODPS) and the Ohio Department of Transportation (ODOT) for the period 2015–2019 [21]. Since this research focuses on motor-vehicle crash severity outcomes induced by drivers aged 65 and older (i.e., senior drivers), we preprocessed the dataset accordingly. We followed the definition of National Highway Traffic Safety Administration to identify senior drivers [22]. We also eliminated crashes such that (1) drivers are aged more than 100; (2) a crash involves more than two vehicles; (3) the cause of collisions is related to commercial vehicles; (4) a crash is pedestrian-involved. Moreover, to specify the risk of senior at-fault crashes to seniors themselves, we distinguish the older driver’s severity from the individual severity information in the dataset and define it as Older driver’s severity. Unlike previous studies that analyzed the maximum severity of a crash, one unique feature of this study is to employ the Older driver’s severity as an outcome variable. 

Each crash severity is recorded by the KABCO injury classification, which is defined as: K—Fatal, A—Incapacitating injury (Serious Injury Suspected), B—Non-incapacitating injury (Minor Injury Suspected), C—Possible injury (Injury Possible), and O—No injury (No Apparent Injury). In this study, the crash severity is aggregated into three classes: Fatal (K + A), Injury (B + C), and PDO (Property Damage Only, O). 

In addition, there is an array of variables in the crash dataset, and we single them out based on our research purpose, a prediction of crash severity by older drivers. The list of our selected predictors is presented in Appendix A Table A1.

### 2.3. Balancing Imbalanced Data

Regarding the classification of the machine learning algorithm, the uneven (imbalanced) class distribution became an important challenge since the 1990s. Within the imbalanced dataset, the specificity or local accuracy of a majority class is greater than that of a minority class. Therefore, “learning from imbalanced data” has initiated in the 2000s [23]. It focuses on how to predict minority classes more accurately by controlling the false positive rate increased. One of the solutions is based on the sampling strategy, which is broadly categorized by undersampling and oversampling [16]. The former (i.e., undersampling) is a sampling approach that reduces the size of a majority class so as to be “balanced” with that of a minority class, whereas the latter (i.e., oversampling) is to duplicate a minority class to increase its size. The undersampling approach has a disadvantage as some valuable information can be lost during its procedure, whereas the oversampling method can result in overfitting as well as an increase of the learning time [16]. Moreover, oversampling minority classes induces the classifiers to focus the specific instances too much, causing generalization problem (The detailed explanation and an example is addressed in Chawla, et al. (2002) [15]). 

In general, the distribution of crash severity is skewed [16]: less severe crashes such as PDO crashes are likely to be in a majority class (having more frequency) while fatal crashes are less prone to occur (belonging to a minority class). When considering the senior driver-involved crashes, severe injury crashes are more important because the older drivers are susceptible to being severely injured [4,6,8,9,10]. However, this imbalanced class distributional feature in the severity of vehicle crashes related to older drivers is yet to be fully addressed in the previous literature. Fiorentini and Losa (2020) [16] reviewed dozens of crash severity prediction studies and investigated distributions of crash severity. They revealed that most recent studies had not employed the balancing methods regardless of the left-skewed class distribution. They addressed that an machine learning algorithm with Random Undersampling the Majority Class (RUMC) outperformed the prediction of the minority class over the original non-balancing machine learning algorithm. 

**Figure 2:**
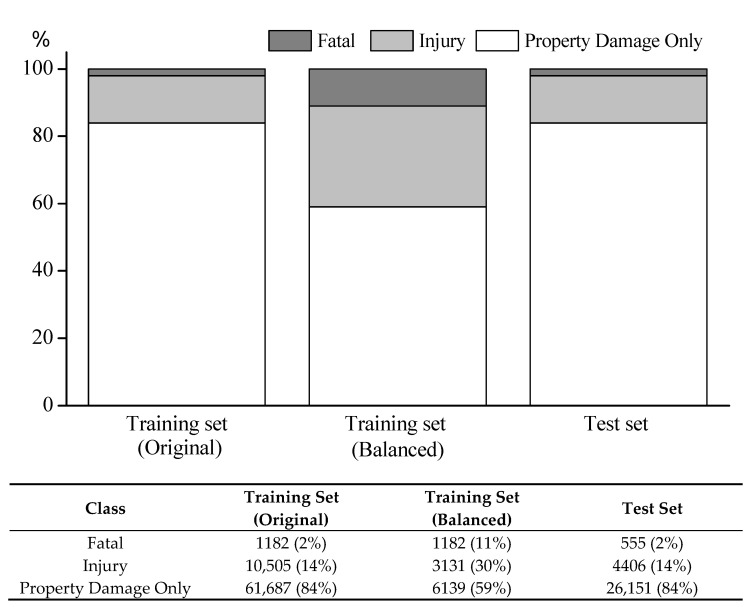
Severity class distribution.

This paper employs the undersampling approach in order to circumvent overfitting and longer duration in learning time. In this setting, all observations of the minority class are kept, while those of other classes are randomly selected and readjusted their size for the sub-training set. We cannot achieve a perfect balance since the sample size of Fatal crashes is too small (i.e., the relative proportion of the Fatal class in the original training set is about 2%, as shown in Figure 2), but mitigate the imbalanced class distribution (i.e., the relative proportion goes up to 11%). The detailed share and frequency distribution of each class for both training and test data sets are provided in Figure 2.

### 2.4. Multinomial Logistic Model (MNL)

Multinomial logit models are traditional discrete outcome models that consider three or more outcomes and do not consider the ordering that may be present in these outcomes. The general framework used to model the degree of injury severity sustained by a crash-involved begins by defining a linear function S that determines the injury severity outcome m for a crash n as

(1) $$S_{mn}=\;\beta_{m}X_{mn}+\varepsilon_{mn}$$

where $\beta_{m}$ is a vector of estimable parameters, $X_{mn}$ is a vector of explanatory variables that are associated with the crash severity m of a crash n, and ε is a disturbance term that accounts for unobserved effects. If the disturbance terms are assumed to be independent and identically distributed (i.i.d.), following generalized extreme value distribution, the multinomial logit model results in [19]:

(2) $$Pr_{n}\left(m\right)=\;\frac{\exp\left(S_{mn}\right)}{\sum_{m}^{3}\exp\left(S_{mn}\right)}=\frac{\exp\left(\beta_{m}X_{mn}\right)}{\sum_{m}^{3}\exp\left(\beta_{m}X_{mn}\right)}$$

The multinomial logit model is susceptible to the correlation of unobserved effects from one injury severity level to the next. Such correlation causes a violation of the model’s independence of irrelevant alternatives (IIA) property. On the plus side, traditional multinomial logistic models do not impose unrealistic parameter restrictions that the conventional ordered probability models do. Further, if the IIA property holds, the model can show that in the presence of underreporting of crashes, all parameters will still be correctly estimated except for the constant term.

We use *multinom* package in R to estimate the generalized multinomial logistic regression model in this study. 

### 2.5. Ordered Logistic Regression (OLR)

Previously emphasized, accounting for the ordinal nature of injury data is an important consideration in crash injury severity modeling. In doing so, traditional ordered probability models have been widely applied [24]. We follow McCullagh’s proportional Odds model (1980) whose link function is the logit [25]. The OLR model is derived by defining a latent variable, S, which is used as a basis for modeling the ordinal ranking of data. The latent variable is specified as:

(3) $$S_{n}=\beta X_{n}+\varepsilon_{n}$$

where $X_{n}$ is a vector of variables determining the discrete ordering for each crash n. β is a vector of estimable parameters, and ε is a random disturbance that is logistically distributed with mean zero (0) and variance one (1). With this observed ordinal-injury data, y, the proportional odds model is

(4) $$log\left(\frac{\mathrm{Pr}(y\leq i|x)}{\mathrm{Pr}(y>i|x)}\right)=\mu_{i}-S_{n}\;(1\leq i<I)$$

With threshold parameters $\mu_{0}<\mu_{1}<\mu_{2}<\ldots <\mu_{i-1}<\mu_{i}$ such that:

(5) $$y_{n}=i\;if\;\mu_{i-1}<S_{n}<\mu_{i}$$

where the coefficients in the latent model and the threshold parameters are estimated using maximum likelihood with the delta method or bootstrapping. The conditional choice probabilities are

(6) $$Pr[y_{n}=m\;|\;X_{n}=x]=\left\{\begin{array}{l} F\left(\mu_{1}-S_{n}\right)\\ F\left(\mu_{m}-S_{n}\right)-F\left(\mu_{m-1}-S_{n}\right)\\ 1-F\left(\mu_{M-1}-S_{n}\right) \end{array}\right\}\begin{array}{l} \;m=1\\ \;1<m<M-1\\ \;m=M \end{array}$$

where the link function F(·) is the logistic cumulative density function. 

Two potential problems potentially arise with a traditional ordered probability approach. First, ordered probability models are susceptible to underreporting of crash-injury data, resulting in biased or inconsistent parameter estimates. If the underreporting rates in the population are known, a weighted maximum likelihood function can be used to analyzed outcome-based samples but the true rate of underreporting is generally unknown, making corrective measures challenging [24,26,27]. In our study, we cannot apply the underreporting rates due to the unavailability of data. The second problem is the restriction in which ordered probability models estimate the parameters of explanatory variables. That is, the estimated effect of an explanatory variable on the outcome variables is consistent [24,26,27]. 

### 2.6. Random Forest (RF)

RF is considered as an ensemble learning method for classification, regression, and other tasks. This method generates many classifiers and aggregates their results. Breiman (2001) [28] proposed an RF as a prediction tool, which consists of a collection of tree-structured classifiers with independent and identically distributed random vectors. For a classification problem, RF constructs a multitude of decision trees and outputs the class which is the major votes of the decision trees. In the decision tree, each node is split using the best one among a subset of predictors randomly chosen at that node. It appears that an RF performed well compared to many other classification models and is less likely to suffer from overfitting issues [28]. Two parameters need to be decided in RF (i.e., the number of trees to grow and the number of variables randomly sampled as candidates at each split). 

Following Lechner and Okasa (2020) [29], RF grows a certain number of decision trees (B) using bootstrapped samples (N) with randomly selected covariates (x). For all B decision trees grown, the conditional mean $E[Y_{n}|X_{n}=x]$ is estimated as the predicted outcome:

(7) $$E[Y_{n}|X_{n}=x]={\hat{RF}}^{B}\left(x\right)=\;\frac{1}{B}\sum_{b=1}^{B}{\hat{T}}_{b}\left(x\right)\;\mathrm{with}\;{\hat{T}}_{b}\left(x\right)=\frac{1}{\left|\left\{n\;:X_{i}\;\in \;L_{b}\left(x\right)\right\}\right|}\sum_{\left\{n\;:X_{n}\;\in \;L_{b}\left(x\right)\right\}}Y_{n}$$

where $L_{b}\left(x\right)$ denotes a leaf containing a predictor x; a tree, ${\hat{T}}_{b}\left(x\right)$ is grown by recursive partitioning until the minimum size is reached. The conditional mean $\;E[Y_{n}|X_{n}=x]$ can be rewritten as follows:

(8) $$E[Y_{n}|X_{n}=x]=\overset{N}{\underset{n=1}{\sum }}{\hat{w}}_{n}\left(x\right)Y_{n}$$

where the weight ${\hat{w}}_{n}$ is defined as an average over every single tree weights ${\hat{w}}_{b,\;n}$:

(9) $${\hat{w}}_{n}\left(x\right)=\frac{1}{B}\overset{B}{\underset{b=1}{\sum }}{\hat{w}}_{b,n}\left(x\right)\;with\;{\hat{w}}_{b,n}\left(x\right)=\frac{1\left\{n\;:X_{n}\;\in \;L_{b}\left(x\right)\right\}}{\left|L_{b}\left(x\right)\right|}$$

Regarding the classification problem in this study, the multinomial random forest estimation procedure follows:

1. Convert categorical severity outcome into dummy variables such as
  (10) $$Y_{m,n}=1\;\left(Y_{n}=m\right)\;for\;m=1,\;2,\;3$$
2. Estimate regression random forests for each dummy variable.
3. Calculate predictions for three regression random forests.
  (11) $${\hat{Y}}_{m,n}=P\left[{\hat{Y}}_{m,n}=1|X_{i}=x\right]=\sum_{n}^{N}{\hat{w}}_{m,n}\left(x\right)Y_{m,n}\;for\;m=1,\;2,\;3$$
4. Compute probabilities for each class
  (12) $${\hat{P}}_{m,n}=\frac{{\hat{Y}}_{m,n}}{\sum_{m=1}^{3}{\hat{Y}}_{m,n}}\;for\;m=1,\;2,\;3$$

Equation (11) defines the probabilities of all three classes and the subsequent Equation (12) represents that the sum of all probabilities equals 1 [29].

In this study, the two hyperparameters are chosen using 10-fold cross-validation (CV) goodness-of-fit of the models. We evaluated different values of the number of trees (*ntree*) as 500, 700, 1000, and 1200. We selected *ntree* = 500 because the accuracies do not improve. We also tested a set of different numbers of (randomly sampled) input factors (*mtry*) (i.e., 13, 15, 17, 19, 21), choosing *mtry* = 13 based on the accuracy. It turned out that the 10-fold CV of the ordered RF results in the same parameters as unordered RF. For the comparison purpose across RF models, we use the same parameters to check their predictive performances. 

### 2.7. Ordered Random Forest (ORF)

ORF is a further extension of RF that is to estimate the ordered choice models with large-dimensional predictors [28,29]. The interest of ORF is directed to the estimation of cumulative probabilities. Lechner and Okasa (2020) [29], who invented an ORF algorithm, present the estimation procedure of ORF as follows:

1. Convert categorical outcome into dummy variables such as
  (13) $$Y_{m,n}=1\;\left(Y_{n}=m\right)\;\mathrm{for}\;m=1,\;2$$
2. Estimate regression RF for each of the M−1 indicators.
3. Calculate probabilities of the class outcome
  (14) $${\hat{Y}}_{m,n}=P\left[{\hat{Y}}_{m,n}=1|X_{n}=x\right]=\sum_{n}^{N}{\hat{w}}_{m,n}\left(x\right)Y_{m,n}\;\mathrm{for}\;m=1,\;2$$
  (15) $${\hat{Y}}_{m,n}=1\;\mathrm{for}\;m=3$$
4. Compute probabilities ${\hat{P}}_{m,n}^{*}$ for each class
  (16) $${\hat{P}}_{m,n}^{*}={\hat{Y}}_{m,n}-{\hat{Y}}_{m-1,n}\;\mathrm{for}\;m=2,\;3$$
  (17) $${\hat{P}}_{m,n}^{*}={\hat{Y}}_{m,n}\;\mathrm{for}\;m=1$$
  (18) $${\hat{P}}_{m,n}^{*}=0\;\mathrm{if}\;{\hat{P}}_{m,n}<0$$
5. Finally, the normalized probabilities ${\hat{P}}_{m,n}$ for each class is given by:
  (19) $${\hat{P}}_{m,n}=\frac{{\hat{P}}_{m,n}^{*}}{\sum_{m=1}^{M}{\hat{P}}_{m,n}^{*}}\;\mathrm{for}\;m=1,\;2,\;3$$

## 3. Results and Discussion

### 3.1. Performance Metrics

Of various performance measurements of the efficacy of learning algorithms, this study employs the performance metrics based on the confusion matrix.

(20) $$\mathrm{Accuracy}=\frac{\mathrm{TP}+\mathrm{TN}}{\mathrm{TP}+\mathrm{FP}+\mathrm{TN}+\mathrm{FN}}$$

(21) $$\mathrm{Precision}=\frac{\mathrm{TP}}{\mathrm{TP}+\mathrm{FP}}$$

(22) $$\mathrm{True}\;\mathrm{Positive}\;\mathrm{Rate}\;\left(\mathrm{TPR}\right)\;\mathrm{or}\;\mathrm{Recall}=\frac{\mathrm{TP}}{\mathrm{TP}+\mathrm{FN}}$$

(23) $$\mathrm{False}\;\mathrm{Positive}\;\mathrm{Rate}\;\left(\mathrm{FPR}\right)=\frac{\mathrm{FP}}{\mathrm{TN}+\mathrm{FP}}$$

(24) $$\mathrm{True}\;\mathrm{Negative}\;\mathrm{Rate}\;\left(\mathrm{TNR}\right)=\frac{\mathrm{TN}}{\mathrm{TN}+\mathrm{FP}}$$

(25) $$F_{1}=\frac{\mathrm{TP}}{\mathrm{TP}+\;\frac{\mathrm{FN}+\mathrm{FP}}{2}}$$

where TP, TN, FP, and FN refer to the number of True Positive instances, True Negative instances, False Positive instances, and False Negative instances, respectively.

It is important to note how each performance measure is linked to prediction performance and efficiency. Accuracy represents how a model can correctly predict the outcomes as much as possible no matter what they are. Accuracy is generally associated with a loss function of machine learning. As a result, conventional machine learning algorithms tend to classify the majority class (PDO). However, Accuracy may not be a proper performance measure if we need to breakdown the predictive performance by severity. 

Other measures such as precision, TPR, and FPR will serve as class-specific performance metrics. Therefore, this study will employ and compare in order to evaluate the influences of the balancing strategy and implementing ordered nature.

Precision, which is defined as the probability of being correctly predicted crash severity, indicates the reliability of the predicted crash severity. For example, suppose that model A has classified 100 fatal crashes, and only 10 crashes are correctly predicted, while model B correctly predicts 50 fatal crashes out of 100. Model B may help, for example, efficiently assigning the resources of emergency medical rescue because of false alarm reduction. 

TPR or recall, which is calculated as the ratio of the number of the correctly predicted to the number of the observed, represents the capability capturing crashes under a certain severity as many as possible. Suppose that there are 1000 observed fatal crashes and model C can detect 100 fatal crashes correctly, whereas model D can find 50 fatal crashes. Then, model C has a higher TPR, which implies that regardless of the reliability (precision), we can discover more fatal crashes and possibly save more lives even though we might spend more resources on false alarms. 

FPR is the risk of models misclassifying the severity of a crash. For example, an FPR on PDO refers to the probability that misclassifies a severer crash as a PDO crash. It is also regarded as the risk of misclassification. A higher FPR on PDO crashes gives rise to the situation that is likely to miss an opportunity to save lives (i.e., risk) as severer crashes are misidentified as PDO. 

In this study, we focus on Accuracy, Precision, TPR, and FPR measures. TNR is [1−FPR] and F1-score is the average between Precision and TPR. According to the previous literature, imbalanced models are apt to predict majority classes (i.e., PDO) while the predictive performance on severer crashes is weak [13,15]. Thus, it can be inferred that as compared to Balanced models, Imbalanced models will have higher overall Accuracy and class-specific performance metrics for PDO, such as TPR. Moreover, Precisions on Fatal crashes of Imbalanced models will be higher than those of Balanced models. As Imbalanced models are likely to focus on identifying PDO when a Fatal crash has at least similar characteristics with PDO, they will classify it as PDO. Then, for remaining Fatal crashes whose characteristics are very Fatal-like, the algorithms will classify them as Fatal. Therefore, the predicted Fatal crashes by Imbalanced models are more likely to be indeed Fatal. 

Conversely, Balanced models are prone to focus on as well as identifying the minority classes (Fatal). Hence, Balanced models are assumed to have lower overall Accuracy and TPR on PDO, higher Precision on PDO, and TPR on Fatal. This indicates that there is a trade-off using a balancing strategy [13,15]. Balanced models can classify in favor of minority classes at the expense of a decrease in the reliability of prediction. Moreover, the overall prediction Accuracy is leveled down.

Although theories and previous literature explain the relationship between crash severity prediction performance metrics and balancing strategy, we attempt to investigate the influence of it on parametric, and non-parametric and ordered, and unordered models. Moreover, the elderly driver’s crash severity should be predicted accurately and efficiently, overcoming two conventional modeling problems. Therefore, based on the preliminaries, we present the outcomes of the analysis, including the predictive performances of ordered models and the influence of balancing strategy on various models in the following sub-sections. 

### 3.2. Overall Predictive Performance

Table 1 presents the predictive performances of our test set using the selected eight classifiers. As an initial step, based on the original training set (that are imbalanced), we implement pairwise comparisons with logistic regression models (i.e., MNL versus OLR) and ensemble-based learning algorithms (i.e., RF versus ORF). Then, we analyze how each classifier performs under different settings of training sets. 

The outcome shows that prediction accuracies range from 81.81% (Balanced OLR) to 86.04% (Imbalanced ORF). When considering the prediction accuracy as a sole model selection criterion, Imbalanced ORF appears to be the best classifier (86.04%) followed by Imbalanced RF (85.91%) and Imbalanced MNL (85.87%), although differences of Accuracy across models are negligible. Overall, the prediction accuracies of Imbalanced models turned out to be superior to those of Balanced models. However, it is worth noting that the prediction accuracy measure may not clearly reflect the ability to predict the crash severity for an imbalanced dataset [16]. Hence, we attempt to further examine various predictive performance metrics so as to have an improved understanding among each classifier. 

### 3.3. Imbalanced Unordinal Predictive Models

Referring to the result of Imbalanced models (i.e., we specifically focus on the unordinal models, MNL and RF) in Table 1, we can observe that prediction accuracies are relatively stable (from 85.87% (MNL) to 85.91% (RF)). This indicates that, without any implementation of ordinal nature and balancing strategy, RF can correctly predict the older driver’s crash severity by 85.91%.

When delving into other metrics, the predictive performance varies with respect to each severity level. For example, if we focus on the precision measure by severity, the imbalanced RF (88.91%) shows the best performance on PDO crashes compared to the imbalanced MNL (88.74%). For Injury and Fatal crashes, precisions by the imbalanced RF are 54.91% to 60.00%, respectively, suggesting that the Imbalanced RF under the precision criterion outperforms the imbalanced MNL. Once we look into the confusion matrix of the classifier (Table 2), however, only 10 crashes are classified as Fatal (while there are 555 Fatal crashes observed), and of 10 Fatal crashes, 6 are correctly predicted. Thus, we can infer that under the imbalanced class distribution, RF (along with MNL) rarely classifies Fatal crashes as Fatal, but the classified Fatal crashes are highly likely to be indeed Fatal (6/10 = 60.00%). 

Under the TPR standard, PDO and Fatal crashes are well classified by MNL (96.63%, and 1.26%, respectively), whereas Injury is detected well by RF (34.16%). While there are slight differences in performance between MNL and RF, these classifiers are weak in predicting minor classes with imbalanced class settings. Unlike TPR of PDO class ranges from 96.42% to 96.63%, that of Injury and Fatal crashes ranges from 34.16% to 32.64%, and 0.90% to 1.26%, respectively. 

Regarding FPR, the best models are MNL on PDO (64.60%) and Injury (4.43%), and RF on Fatal (0.01%) classes. That is, assuming that an ambulance does not go out for a predicted PDO crash the Imbalanced MNL classifier, there is 64.60% chance that the predicted PDO crashes are indeed severer crashes so as that an ambulance should have been out. 

In short, an investigation of the predictive performance by confusion matrix suggests that the imbalanced distribution of classes affects the predictive capabilities of the unordinal classifiers. Stated differently, the unordinal classifiers would predict most crashes as PDO and/or Injury rather than Fatal under imbalanced datasets. This leads us to further explore the influence of balanced approaches, such as an undersampling strategy. 

### 3.4. Handling Class Imbalanced Distribution

The balancing strategy pays attention to the minor classes. In this section, we examine how balancing the skewed distribution of outcome classes affects the predictive performance. Table 3 presents changes in predictive performances by using balanced classifiers. It is worth noting that there are pros and cons of balancing class distributions. 

First of all, we have analyzed the individual predictive performances of the balanced classifiers. ORF shows the best overall predictive accuracy (83.57%), followed by MNL (82.35%), RF (81.85%), and OLR (81.81%). With respect to precision, ORF demonstrates the best performance in predicting Injury and Fatal crashes (44.95% and 30.57%), while RF outperforms other counterparts in the case of PDO crashes (91.87%). Considering TPR, ORF classifies PDO crashes (90.89%) the best, while RF and OLR perform better in predicting Injury and Fatal crashes (52.07% and 25.41%), respectively. In addition, FPRs of each model reveal that RF (41.18%), ORF (9.92%), and ORF (0.52%) models are the best ones for PDO, Injury, and Fatal crashes classification, respectively.

Comparison of prediction performance metrics between Imbalance and Balance models suggests that models using a balancing strategy are more likely to identify minor classes such as Injury and Fatal. This implies that classifiers utilizing a balancing strategy can significantly detect more severe crashes than their imbalanced counterparts. For instance, TPRs of Injury and Fatal categories by means of the Balanced ORF are improved by 13.8% (from 35.27% to 49.07%) and 11.71% (from 0.90% to 12.61%), respectively (One may refer to Table 1 and Table 3 for details). 

In terms of precisions, all Balanced models perform better than Imbalanced models in predicting PDO cases. In contrast, precision metrics for Injury and Fatal crashes under imbalanced data show better predictive performance (e.g., 55.44% versus 44.95% for Injury and 62.50% versus 30.57% for Fatal by ORF as depicted in Table 1). We argue that one should interpret these results with care as the predicted frequency of minority classes tends to be very small so as to inflate the magnitude of precision. For example, the Balanced ORF model predicts 229 crashes as Fatal crashes and correctly classified 70 out of 555 Fatal crashes (Table 4), while the Imbalanced ORF model predicts detect only 8 Fatal crashes and 5 crashes are indeed Fatal (Table 5). Unfortunately, it is still uncertain whether the predicted severe classes by the Balanced classifiers belong to those classes due to the lower precision. 

Meanwhile, a balancing strategy results in weaker global prediction capability. Table 4 shows that the prediction accuracies of balanced classifiers are dropped from 2.47% (ORF) to 4.06% (RF). FPRs on PDO with the balanced set have decreased by 15.88–22.21%, whereas those on Injury and Fatal crashes have increased by 0.51–7.62%. This indicates that the risk of erroneously predicting severer crashes as PDO decreases, and, at the same time, the risk of misclassifying PDO into severer crashes increases. Therefore, we can confirm that models based upon the balancing method are in favor of minority classes at the expense of misclassifying some major classes. 

### 3.5. Implementing Ordinal Nature into Models

The outcome of crash severity prediction models is ordered. Amemiya (1985) [18] argues that taking the ordered nature of the categorical response variable into consideration can improve the efficiency of the model. Meanwhile, ordered models cannot always be superior to unordered counterparts due to varying influences of predictors on outcome probabilities [19]. For example, safety equipment such as an airbag can reduce the probability of fatal crashes but instead increase that of severe injury crash because an airbag would only be activated in a deadly accident. In this scenario, the parameter *airbag* in an unordered model may be positive and negative for severe and fatal injury outcomes, respectively. However, the parameter in an ordered model would be inappropriate. Put differently, a tradeoff is inherently being made between recognizing the ordering of responses and losing the flexibility in specification offered by unordered models [19]. Table 6 shows the differences in predictive metrics between ordered and unordered classifiers. The Balanced ORF approaches are mostly superior to others, while the effects tend to vary across performance measures.

According to Table 6, ORF outperforms RF by 1.73% in terms of accuracy, showing improvement in the overall predictive performance. Considering the other metrics, accounting for an ordinal nature into the modeling process has shown significant improvements or dis-improvements for Balanced models while it appears no significant differences for Imbalanced models. For instance, we find that the differences of predictive metrics among Imbalanced models are relatively negligible across all severity levels (i.e., −1.18–2.50%). The Balanced ORF is improved and dis-improved significantly in precision and TPR of severer crashes from the Balanced RF. Precisions of Injury and Fatal increase by 3.73% and 8.24%, respectively, with a decrease in that of PDO (−0.70%). FPRs of Injury and Fatal decrease by 2.33% and 0.57%. This implies that the advantage of employing an ordinal nature into the Balanced RF is that predicted Injury and Fatal crashes are more likely to be correct. The downside of it, however, is that TPRs of Injury and Fatal decrease by 3.00% and 4.68%, respectively. That is, the Balanced ORF cannot find severer crashes as good as the Balanced RF can. 

### 3.6. Influential Factors

Table 7 lists the top ten influential factors identified by each classifier. We have selected the influential variables of MNL and OLR based on t-statistics. A decrease of Gini Impurity is adopted to choose influential factors in the case of RF and ORF. Predictors such as *Airbag* and *Unit speed*, followed by *same direction (Manner of collision)*, *Safety Equipment*, and *Number of units* are found to be frequent as well as influential across classifiers. We verify that the Imbalanced MNL classifiers have a different set of influential factors compared to the rest. This may be because of its different estimation procedure of MNL, unlike OLR whose parameters of explanatory variables are constrained to be consistent across each severity level (thus, OLR produces a single set of estimated coefficients). When it comes to RF and ORF, influential factors are aggregated over all severity levels. 

Regarding important predictors, such as *Airbag*, *Unit speed*, *Safety Equipment*, and *Number of units*, we have analyzed their associations with crash severity. Table 8 presents a cross-tabulation of *Airbag* variable in response to each severity, revealing that the relative proportion of *Airbag* use becomes lower alongside an increase of severity levels. The Pearson’s Chi-squared test supports that the usage of airbag is not independent of the severity of crashes with strong statistical significance (χ^2^ = 6281, df = 2, *p*-value < 0.0001). Likewise, the percentage of using safety equipment becomes lower as severity increases (χ^2^ = 762.6, df = 2, *p*-value < 0.0001). 

A cross-tabulation of *number of units* variable shows that the more vehicle units are involved in a crash, the more it results in a severer one. The relative proportion of PDO crashes involving more than 3 units is 4.7%, while that of Injury and Fatal crashes is 10.24% and 12.07%, respectively (see the last row of Table 8). 

**Figure 3:**
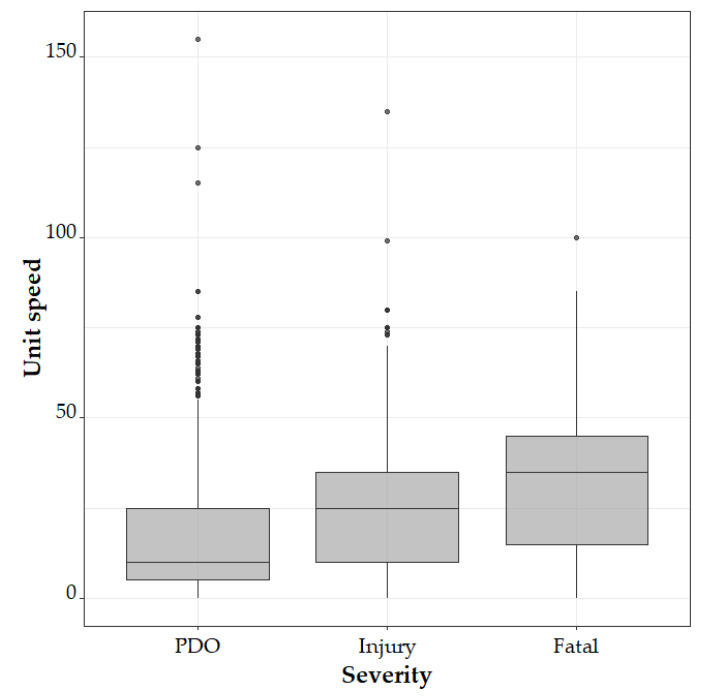
Boxplot of unit speed.

The association between crash severity and *unit speed* is investigated. Figure 3 shows that a unit speed is positively associated with crash severity (i.e., higher unit speeds are more likely to result in severer outcomes on average), even though there are few outliers in PDO category (high-speed driven PDO cases). The difference in unit speed regarding severity is also confirmed via a pairwise t-test adjusted by the Benjamini–Hochberg method [30], suggested by strong statistical significance in Table 9.

## 4. Discussion

Along with demographic transition characterized by population aging, senior-involved vehicle crashes gain broader attention than before. Although there appears to be a decreasing trend in the number of senior-related crashes, the risk of injury has been elevated. Due to senior involvement and their physical vulnerability, predicting severe senior-involved crashes becomes more important than other types of crashes. The challenge is, however, how to adequately cope with an imbalanced severity class distribution and ordered nature of crash severities, as these can complicate the classification of senior-involved crashes. Thus, we suggest implementing ordered nature and handling imbalanced crash severity to improve the prediction of the severity of crashes by older drivers. Adopting machine learning algorithms including logistic models and random forests (each pair of ordered and unordered responses), we attempt to compare their predictive performance underbalanced and/or imbalanced class settings. The key findings of this study are explained below.

Many studies have considered a two-level crash severity (i.e., crashes other than PDO aggregated to Injuries). This is because they have not handled a class imbalance problem so that they failed to achieve a proper size of observations for minor classes such as fatal crashes. This aggregation inevitably results in a loss of information, which might hamper predicting detailed crash severity. Few studies have tried to overcome this issue by introducing a sampling strategy. Still, there is a lack of works attempting to implement the ordered nature in the model as well as handling the imbalanced classification. 

Using Ohio vehicle crash data, we learn, test, and compare the performance of multiple predictive models to evaluate the effects of implementing the ordered nature and handling imbalanced class. The results of performance metrics and confusion matrices are as follows: firstly, without any implementation of ordinal nature and balancing strategy, the multinomial logistic and random forest models show acceptable predictive performance in terms of prediction accuracy. The performances in predicting minority class, however, are poor since the low TPRs of Injury and Fatal crashes and the high FPR of PDO crashes. This implies that the Imbalanced models cannot find the Injury and Fatal crashes enough so as to classify most severer crashes into PDO. Thus, there is a high risk of error to overlook severer crashes. 

Secondly, employing a balancing strategy enhances performances in predicting severer crashes. TPRs on Injury and Fatal crashes in the Balanced models are higher than those of the Imbalanced classifiers (by 11.71–23.96%). Moreover, the risk of misclassifying severer crashes as PDO decreases. However, there is a compromise as well. The reliability in predicting severer crashes is still in question as FPRs on Injury and Fatal in the Balanced models decrease. The reason might be a loss of information or the traits of cost-sensitive analysis. Further research is required. 

Nonetheless, we can take practical advantages from the Balanced models. For example, once a senior driver-related crash occurs and its information is achieved by police and hospital, they can expect the degree of injury in order to assign resources appropriately, such as an ambulance and an emergency helicopter. Furthermore, an insurance company can utilize this model to prevent insurance fraud. 

Third, implementing ordered nature on logistic regression models does not significantly improve the predictive performances across all severer levels, while prediction accuracy and precision of ordered random forest algorithm have improved as compared to unordered random forest. This suggests that, as mentioned by Washington et al. (2020) [19], the relationships between predictors and crash severity outcomes might not be monotonous. Our study might be the case as the predictive capabilities of ordered random forest models were enhanced. 

Additionally, it turns out that the effects of a balancing strategy for ordinal models are not significantly different from those of multinomial models. There is no best model, but we can utilize some models according to the purpose. For overall prediction performance, the Imbalanced ORF classifier is the best. If the major focus is on predicting fatal crashes as much as possible, we can use the Balanced OLR (the highest TPR on Fatal). If we intend to predict fatal crashes in a precise way to avoid the cost from incorrectly identifying fatal crashes, we may go with the Balanced ORF model (the highest precision on Fatal). Since countless ML algorithms are utilized to predict crash severity, we cannot determine that any specific algorithms are the best. Rather, we hereby address that implementing the balancing strategy on any models (un- and ordered/parametric or non-parametric models) can enhance the minority class-specific prediction performance, while the effects of operating ordered nature are inconclusive (Table 10).

The analysis of the top ten influential factors reveals the important predictors of senior driver’s injury (i.e., *Airbag*, *Unit speed*, *Safety Equipment*, and *Number of units*, and *same direction (manner of collision)*). What emerges from the list of the influential factors is that the MNL has a different set of influential factors compared to the other models. It will be interesting to investigate why the imbalanced MNL acts differently in predicting influential factors in future research. Additionally, we might be able to develop a way to represent the standardized influential factors with respect to each level employing sensitivity analysis, e.g., see, for example, X. Li, Lord, Zhang, and Xie, (2008), Z. Li, Liu, Wang, and Xu (2012), Yu and Abdel-Aty (2013) [31,32,33].

## 5. Conclusions

There are several limitations that we fail to address in this study. Since we cannot achieve any statistical inference of the performance metrics, it should be noted that the differences or superiorities of the predictive performance of each algorithm may not be applicable to other data or cases. For future research, we may consider running the Monte Carlo simulation to have statistical inferences of performance metrics. Even if the minor class-specific prediction performances are improved significantly, we still need to improve the model fits for practical use. For example, the best algorithm of TPR on Fatal accidents is 25.41% under the ordered logit model. This means that approximately 75% of Fatal crashes are yet to be overlooked. Moreover, the unstable manifest of influential predictors may be due to the low model fit.

Despite analyzing the influential predictors across algorithms, we could not shed light on how various ML algorithms recognize those predictors differently in order to classify crash severity. In future research, we can investigate which fatal crashes are likely to be correctly classified by Balanced models but not by Imbalanced models. We operated a balancing strategy for the imbalanced class distribution by an undersampling method. As a result, the sample sizes between Balanced and Imbalanced models are different. This can affect the prediction performance. The overall Accuracies of Balanced models are lower than their counterparts because the sample size of Balanced dataset is smaller. We may consider equalizing the sample size utilizing any advanced method (e.g., Synthetic Majority Oversampling TEchnique, SMOTE) for future research.

Regardless of the limitations, this study can provide a stepping stone for developing a more efficient traffic injury predictive model for older drivers by balancing technique and accounting for an ordinal nature. We believe our approach allows us to improve our understanding of severity of crashes induced by older drivers, which leads to enhancing public safety and health.

## Figures and Tables

**Table 1:** Predictive performance of the selected eight models on the test set.

Performance Metrics	Models							
	Imbalanced				Balanced			
	MNL ^1^	OLR ^1^	RF ^1^	ORF ^1^	MNL	OLR	RF	ORF
**Accuracy**	85.87%	85.86%	85.91%	86.04%	82.35%	81.81%	81.85%	83.57%
**Precision**								
PDO ^2^	88.74%	88.77%	88.91%	89.07%	91.52%	91.65%	91.87%	91.16%
Injury	54.89%	54.66%	54.91%	55.44%	42.16%	40.30%	41.22%	44.95%
Fatal	41.18%	40.00%	60.00%	62.50%	23.77%	22.24%	22.33%	30.57%
**TPR ^3^**								
PDO	96.63%	96.62%	96.42%	96.40%	89.27%	88.93%	88.23%	90.89%
Injury	32.64%	32.61%	34.16%	35.27%	49.16%	46.66%	52.07%	49.07%
Fatal	1.26%	1.44%	1.08%	0.90%	20.00%	25.41%	17.30%	12.61%
**FPR ^3^**								
PDO	64.60%	64.42%	63.39%	62.33%	43.62%	42.73%	41.18%	46.44%
Injury	4.43%	4.46%	4.63%	4.68%	11.12%	11.41%	12.25%	9.92%
Fatal	0.03%	0.04%	0.01%	0.01%	1.17%	1.61%	1.09%	0.52%
**TNR ^3^**								
PDO	35.40%	35.58%	36.61%	37.67%	56.38%	57.27%	58.82%	53.56%
Injury	95.57%	95.54%	95.37%	95.32%	88.88%	88.59%	87.75%	90.08%
Fatal	99.97%	99.96%	99.99%	99.99%	98.83%	98.39%	98.91%	99.48%
**F_1_^3^**								
PDO	73.98%	74.03%	74.22%	74.52%	76.66%	76.78%	76.92%	76.59%
Injury	47.62%	47.59%	49.22%	50.40%	61.34%	59.04%	63.37%	61.73%
Fatal	2.49%	2.84%	2.14%	1.79%	33.01%	40.00%	29.22%	22.30%

Note. ^1^ Models: MNL = Multinomial logistic regression; OLR = Ordinal logistic regression; RF = Random Forest; ORF = Ordered Random Forest; ^2^ PDO = Property Damage Only; ^3^ TPR = True Positive Rate; FPR = False Positive Rate; TNR = True Negative Rate; F_1_ = F_1_ score.

**Table 2:** Confusion matrix of multinomial logistic model (MNL)-imbalanced model.

		Observed			Total (Predicted)
		PDO	Injury	Fatal	
**Predicted**	**PDO**	25,216	2897	248	28,301
	**Injury**	935	1505	301	2803
	**Fatal**	0	4	6	8
	**Total (observed)**	26,151	4406	555	

**Table 3:** Improved predictive performance by using a balancing strategy.

Model	Logistic Model		Random Forest	
	Multinomial	Ordered	Multinomial	Ordered
**Accuracy**	−3.52%	−4.05%	−4.06%	−2.47%
**Precision**				
PDO	2.77%	2.87%	2.96%	2.09%
Injury	−12.72%	−14.36%	−13.69%	−10.49%
Fatal	−17.41%	−17.76%	−37.67%	−31.93%
**TPR**				
PDO	−7.36%	−7.69%	−8.19%	−5.51%
Injury	16.52%	14.05%	17.91%	13.80%
Fatal	18.74%	23.96%	16.22%	11.71%
**FPR**				
PDO	−20.98%	−21.69%	−22.21%	−15.88%
Injury	6.70%	6.94%	7.62%	5.24%
Fatal	1.13%	1.57%	1.08%	0.51%
**TNR**				
PDO	20.98%	21.69%	22.21%	15.88%
Injury	−6.70%	−6.94%	−7.62%	−5.24%
Fatal	−1.13%	−1.57%	−1.08%	−0.51%
**F_1_**				
PDO	2.68%	2.75%	2.70%	2.08%
Injury	13.72%	11.46%	14.15%	11.32%
Fatal	30.52%	37.16%	27.08%	20.51%

**Table 4:** Confusion matrix of ORF-balanced model.

		Observed			Total (Predicted)
		PDO	Injury	Fatal	
**Predicted**	**PDO**	23,769	2148	156	26,073
	**Injury**	2319	2162	329	4810
	**Fatal**	63	96	70	229
	**Total (observed)**	26,151	4406	555	

**Table 5:** Confusion matrix of ORF-imbalanced model.

		Observed			Total (Predicted)
		PDO	Injury	Fatal	
**Predicted**	**PDO**	25,209	2849	243	28,301
	**Injury**	942	1554	307	2803
	**Fatal**	0	3	5	8
	**Total (observed)**	26,151	4406	555	

**Table 6:** The difference of predictive performances between ordered and multinomial models.

Model	Imbalanced Model		Balanced Model	
	Logistic	Random Forest	Logistic	Random Forest
**Accuracy**	−0.01%	0.13%	−0.54%	1.73%
**Precision**				
PDO	0.03%	0.16%	0.13%	−0.70%
Injury	−0.23%	0.53%	−1.87%	3.73%
Fatal	−1.18%	2.50%	−1.53%	8.24%
**TPR**				
PDO	−0.01%	−0.03%	−0.34%	2.66%
Injury	−0.02%	1.11%	−2.50%	−3.00%
Fatal	0.18%	−0.18%	5.41%	−4.68%
**FPR**				
PDO	−0.18%	−1.07%	−0.89%	5.26%
Injury	0.04%	0.05%	0.28%	−2.33%
Fatal	0.01%	0.00%	0.45%	−0.57%
**TNR**				
PDO	0.18%	1.07%	0.89%	−5.26%
Injury	−0.04%	−0.05%	−0.28%	2.33%
Fatal	−0.01%	0.00%	−0.45%	0.57%
**F_1_**				
PDO	0.05%	0.29%	0.11%	−0.33%
Injury	−0.04%	1.18%	−2.30%	−1.64%
Fatal	0.35%	−0.35%	6.99%	−6.92%

**Table 7:** Comparison of influential factors.

	MNL				OLR		RF		ORF	
	Imbalanced		Balanced		Imbalanced	Balanced	Imbalanced	Balanced	Imbalanced	Balanced
	Injury	Fatal	Injury	Fatal						
1	Slush (RC)	Rear-to-rear (MC)	Sand;Mud; Dirt;Oil;Gravel (RC)	Vision Obstruction (CC)	**Airbag**	**Airbag**	**Airbag**	**Airbag**	**Airbag**	**Airbag**
2	DD_drv	Improper Crossing (CC)	**Airbag**	Improper Crossing (CC)	**Safety Equipment**	**Safety Equipment**	**Unit Speed**	**Unit Speed**	**Unit Speed**	**Unit Speed**
3	Posted Speed	Stopped or Parked Illegally (CC)	**Unit Speed**	Dirt (RS)	**Unit Speed**	**Unit Speed**	Posted Speed	**Number of Units**	Posted Speed	**Number of Units**
4	Passenger Van (UT)	Dirt **(RS)**	**Male**	Stopped or Parked Illegally (CC)	**Male**	Sideswipe; same direction (MC)	**Number of Units**	Posted Speed	**Number of Units**	Posted Speed
5	Blowing Sand; Soil; Dirt; Snow (Weather)	Vision Obstruction (CC)	Sideswipe; same direction (MC)	Rear-to-rear (MC)	Sideswipe; same direction (MC)	**Male**	**Male**	Sideswipe; same direction (MC)	Following too Close/ACDA (CC)	Not Collision (MC)
6	**Airbag**	**Airbag**	**Safety Equipment**	**Airbag**	Making Right Turn (PA)	**Age**	Following too Close/ACDA (CC)	**Safety Equipment**	Not Collision (MC)	Sideswipe; same direction (MC)
7	Number of Occupants	**Safety Equipment**	**Age**	**Safety Equipment**	**Age**	Changing Lanes (PA)	Sideswipe; same direction (MC)	Not Collision (MC)	**Male**	Striking (Action)
8	Making U-Turn (PA)	Freezing Rain or Drizzle (Weather)	Making Right Turn(PA)	**Unit Speed**	Changing Lanes (PA)	Posted Speed	LocationRoadType_NA	Striking (Action)	Sideswipe; same direction (MC)	Angle (MC)
9	Driverless (PA)	**Unit Speed**	Small Truck Related	Posted Speed	Not Collision (MC)	Making Right Turn (PA)	Not Collision (MC)	Other Improper Action (CC)	LocationRoadType_NA	LocationRoadType_NA
10	YouthRelated	**Male**	Changing Lanes (PA)	Age	Other Improper Action (CC)	Head-on (MC)	RoadwayDivided	Following too Close/ACDA (CC)	Angle (MC)	Following too Close/ACDA (CC)

Note. (1) Road condition = RC; Road surface = RS; Manner of collision = MC; Precrash action = PA; Contribution circumstance = CC; Unit type = UT; Not Collision = Not Collision Between Two Vehicles in Transport. (2) Commonly Influential factors across different learning algorithms (classifiers) are highlighted **in bold**.

**Table 8:** Instances and percentage of crash severity by influential predictors.

		PDO	Injury	Fatal
Airbag	Not	24,591 (94.03%)	2472 (56.11%)	214 (35.26%)
	Used	1560 (5.97%)	1934 (43.89%)	341 (61.44%)
Safety Equipment	Not	403 (1.54%)	185 (4.20 %)	99 (17.84%)
	Used	25,748 (98.46%)	4221 (95.80 %)	456 (82.16%)
Number of units	1	2790 (10.67%)	1163 (26.40%)	190 (34.23%)
	2	22,131 (84.63%)	2792 (63.37%)	298 (53.69%)
	3	1101 (4.21%)	372 (8.44%)	53 (9.55%)
	4	105 (0.40%)	66 (1.50%)	8 (1.44%)
	5	22 (0.08%)	8 (0.18%)	5 (0.90%)
	6	1 (0.00%)	3 (0.07%)	1 (0.18%)
	7	1 (0.00%)	1 (0.02%)	0 (0.00%)
	8	0 (0.00%)	1 (0.02%)	0 (0.00%)
	3–8	1230 (4.70%)	451 (10.24%)	67 (12.07%)

**Table 9:** Pairwise comparison by *t*-test.

Unit Speed	PDO	Injury	Fatal
Mean	17.61	25.97	31.17
Standard deviation	15.51	17.47	19.45
Pairwise comparison using *t*-test (adjusted by Benjamini–Hochberg method)		*p* < 0.001	*p* < 0.001

**Table 10:** Application of strategies depending upon purposes.

**Strategy**	**Purpose**	
	**Overall Prediction**	**Class-Specific (Minority) Prediction**
Non	Good	Bad
Balanced	Bad	Good
Ordered	Inconclusive	Inconclusive
Balance + Ordered	Bad	Good

## Data Availability

Publicly available datasets were analyzed in this study. The original data can be found here: https://ohtrafficdata.dps.ohio.gov/CrashStatistics/Home. For the research purpose, a series of data manipulation has been conducted. Hence, the data presented in this study are available on request from the corresponding author.

## Appendix A

**Table A1:** The list of variables.

Variables	Type of Factor	Label
Older driver’s severity	Nominal (Ordinal)	Fatal
		Injury
		PDO
Driver age	Numeric	Driver age
Number of occupants	Numeric	Number of occupants
Number of units	Numeric	Number of vehicles involved in a crash
Weather	Nominal	Blowing Sand; Soil; Dirt; Snow
		Clear
		Cloudy
		Fog; Smog; Smoke
		Freezing Rain or Freezing Drizzle
		Other/Unknown
		Rain
		Severe Crosswinds
		Sleet; Hail
		Snow
Light condition	Nominal	Dark—Lighted Roadway
		Dark—Roadway Not Lighted
		Dark—Unknown Roadway Lighting
		Dawn/Dusk
		Daylight
		Other/Unknown
School zone related	Nominal	Active school zone related (1) or not (0)
Work zone related	Nominal	Work zone related (1) or not (0)
Crash action	Nominal	Both striking and struck
		Non-collision
		Non-contact
		Other/Unknown
		Striking
		Struck
Precrash action	Nominal	Backing
		Changing Lanes
		Driverless
		Entering Traffic Lane
		Leaving Traffic Lane
		Making Left Turn
		Making Right Turn
		Making U-Turn
		Negotiating a Curve
		Other/Unknown
		Overtaking/Passing
		Slowing or Stopped In Traffic
		Straight Ahead
Contributing circumstance	Nominal	Drove off Road
		Failure to Yield
		Following too Close/ACDA
		Improper Backing
		Improper Crossing
		Improper Lane Change
		Improper Passing
		Improper Start From a Parked Position
		Improper Turn
		Left of Center
		Load shifting/Falling/Spilling
		Lying in Roadway
		None
		Not Discernible
		Opening Door into Roadway
		Operating Defective Equipment
		Other Improper Action
		Ran Red Light
		Ran Stop Sign
		Stopped or Parked Illegally
		Swerving to Avoid
		Unsafe Speed
		Vision Obstruction
		Wrong Way
Manner of collision	Nominal	Angle
		Backing
		Head-on
		Not Collision Between Two Vehicles in Transport
		Other/Unknown
		Rear-end
		Rear-to-rear
		Sideswipe; opposite direction
		Sideswipe; same direction
Animal related	Nominal	Animal related (1) or not (0)
Motorcycle related	Nominal	Motorcycle related (1) or not (0)
Speed related	Nominal	Speed related (1) or not (0)
Semitruck related	Nominal	Semitruck related (1) or not (0)
Small truck related	Nominal	Small truck related (1) or not (0)
Gender	Nominal	Male (1) or female (0)
Alcohol related	Nominal	Alcohol related (1) or not (0)
Drug related	Nominal	Drug related (1) or not (0)
Youth related	Nominal	Youth related (1) or not (0)
Teen related	Nominal	Teen related (1) or not (0)
DUI21 related	Nominal	DUI21 related (1) or not (0)
Commercial related	Nominal	Commercial related (1) or not (0)
Location road type	Nominal	Highway
		Not highway
		No information
Intersection or approach related	Nominal	Intersection or Approach Related (1) or not (0)
Within interchange area	Nominal	Within Interchange Area (1) or not (0)
Roadway divided	Nominal	Roadway is Divided (1) or not (0)
Road contour	Nominal	Curve Grade
		Curve Level
		Other/Unknown
		Straight Grade
		Straight Level
Road condition	Nominal	Dry
		Ice
		Other/Unknown
		Sand; Mud; Dirt; Oil; Gravel
		Slush
		Snow
		Water (Standing; Moving)
		Wet
Road surface	Nominal	Blacktop; Bituminous; Asphalt
		Brick/Block
		Concrete
		Dirt
		Other/Unknown
		Slag; Gravel; Stone
		Unit Speed
		Posted Speed
Unit type	Nominal	Passenger Car
		Passenger Van (minivan)
		Pick up
		Sport Utility Vehicle
